# Retention through career development: on-the-job training in Trinidad

**Published:** 2018-07-31

**Authors:** Lynn Anderson, Sonja Johnston

**Affiliations:** 1 CEO: International Joint Commission on Allied Health Personnel in Ophthalmology (IJCAHPO), Minnesota, USA.; 2 Consultant Ophthalmologist: Caribbean Eye Institute, Valsayn, Trinidad and Tobago, West Indies.

Recruiting, training and retaining an effective eye care team present challenges for the 35 ophthalmologists working in Trinidad and Tobago due to a shortage of allied ophthalmic personnel (AOP). AOP are essential members of the eye team and support both the productivity and quality of eye care provided.

In order to address the skills shortage, our private eye care practice has developed an on-the-job training (OJT) model that implements the International Joint Commission on Allied Health Personnel in Ophthalmology's (IJCAHPO) curriculum.

Potential candidates are offered a job in a practice (30% in our practice, and 70% in other practices in Trinidad and Tobago) and come to us for a six-month programme of basic to advanced courses. These are delivered by four ophthalmologists from our practice as well as guest lecturers.

Potential candidates have to meet the Caribbean Examination Council's required level of skills (or equivalent) in English, mathematics, science and computer literacy. Students receive textbooks; participate in classroom, skills, independent, and on-line training; and are assessed using quizzes and rubrics. Clinical training provides patient experience. Upon completion, students take certification preparation courses and take the IJCAHPO's certification examinations. The examinations are offered at the Ophthalmological Society of the West Indies’ (OSWI) annual congress and at computer-based centres in Trinidad.

Fifteen allied ophthalmic personnel are currently in training. Long-term programme viability will be enhanced by increasing the number of trainees and by state recognition of the qualification – this will create job opportunities for our trainees in public hospitals. Formal, recognised AOP training programmes will then be sustainable and produce sufficient personnel to ensure the country's workforce is in keeping with the global health workforce requirements.

Training and qualification supports:

- Retention by building technician self-esteem
- Career progression with increased responsibilities and remuneration
- Lateral movement of employees, e.g. from one practice to another or to a government hospital.

At our practice, our training, retention and reward (‘perk’) strategies are aligned, and involve sending employees to conferences locally and abroad and giving them leadership roles. At OSWI's annual conference, we conduct a 4-5 day workshop consisting of lectures and hands-on training which is taught by ophthalmologists and qualified allied ophthalmic personnel.

We also recognise our employees by displaying their diplomas alongside their photographs so that patients can recognise their professional status and afford them the respect they deserve.

